# The feasibility and effectiveness of a streamlined single‐catheter approach for radiofrequency atrial fibrillation ablation

**DOI:** 10.1002/joa3.12390

**Published:** 2020-06-26

**Authors:** Shui Hao Chin, Jim O'Brien, Gianluca Epicoco, Prithvi Peddinti, Akanksha Gupta, Simon Modi, Johan Waktare, Richard Snowdon, Dhiraj Gupta

**Affiliations:** ^1^ Institute of Cardiovascular Medicine and Science Department of Cardiology Liverpool Heart and Chest Hospital Liverpool UK; ^2^ School of Medicine University of Liverpool Liverpool UK; ^3^ School of Medicine Imperial College London UK

**Keywords:** Ablation Index, atrial fibrillation, catheter ablation, CLOSE protocol, pulmonary vein isolation

## Abstract

**Background:**

Catheter ablation for atrial fibrillation (AF) traditionally requires the use of circular mapping catheter (CMC) for pulmonary vein isolation (PVI). This study aimed to assess the feasibility and effectiveness of a CMC‐free approach for AF ablation performed by a contiguous optimized (CLOSE) ablation protocol.

**Methods:**

A CLOSE‐guided and CMC‐free PVI protocol with a single transseptal puncture was attempted in 67 patients with AF. Left atrial (LA) CARTO voltage mapping was performed with the ablation catheter pre‐ and postablation to demonstrate entry block into the pulmonary veins, and pacing maneuvers were used to confirm exit block.

**Results:**

The CMC‐free approach was successful in achieving PVI in 66 (98.5%) cases, with procedure time of 148 ± 32 minutes, ablation time of 27.5 ± 5.7 minutes, and fluoroscopy time of 7.8 ± 1.0 minutes. First‐pass PVI was seen in 58(86.5%) patients, and pacing maneuvers successfully identified the residual gap in eight of the other nine cases. No complication was observed. At 12 months follow‐up, 60 (89.6%) patients remained free from AF. The CMC‐free approach resulted in a cost saving of £47,190.

**Conclusion:**

A CMC‐free CLOSE‐guided PVI approach is feasible, safe, and cost‐saving, and is associated with excellent clinical outcomes at 1 year.

## Graphical abstract

Catheter ablation for atrial fibrillation traditionally requires the use of circular mapping catheter for pulmonary vein isolation. Using a contiguous (CLOSE protocol) and Ablation Index‐guided workflow, this study demonstrated that a single‐catheter approach is feasible, effective and cost‐saving, producing superior first‐pass isolation rate and 12‐month clinical outcome.

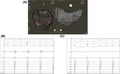

## 1 INTRODUCTION

Electrical isolation of the pulmonary veins (PVs) is the cornerstone of atrial fibrillation (AF) ablation.^1^ The evolutionary displacement of ablation lesion sets from the PV ostia to the left atrial (LA) antra producing a wide antral circumferential ablation has served to increase clinical success and reduce the risk of PV stenosis^1^ at the expense of an increased propensity for gaps along the ablation lesion sets.^2^, ^3^ As such, a circular multipolar catheter has traditionally been employed at the PV ostium to evaluate PV electrical activity and identification of potential gaps in the LA‐PV conduction. The conventional point‐by‐point radiofrequency (RF) ablation setup with multiple catheters residing in the LA is therefore potentially complex, skill‐dependent, time‐consuming and has required double transseptal punctures or single‐puncture double‐wiring transseptal catheterization techniques.

Recently, the use of a weighted formula (Ablation Index, AI), that incorporates power, contact force (CF), and time, has yielded superior rates of durable pulmonary vein isolation (PVI) and freedom from arrhythmia.^4^, ^5^ In addition, use of automated lesion tagging, and adherence to stringent intertag distance (ITD) targets as part of the contiguous lesion optimized (CLOSE) protocol has played an integral role in minimizing gaps along ablation lines.^6^, ^7^, ^8^

With the very high first‐pass PVI rates observed with CLOSE ablation (as high as 97% in our experience^5^), it is debatable whether a circular mapping catheter (CMC) is still needed, but the feasibility of a CMC‐free CLOSE ablation approach has not been studied. In this prospective study, we evaluated the feasibility of a single transseptal CMC‐free approach with contiguous AI‐guided PVI, and assessed the clinical efficacy of this technique over a 12‐month follow‐up period.

## 2 METHODS

### 2.1 Patient population

The study population comprised consecutive patients who underwent first‐time RF PVI for symptomatic drug‐refractory AF at our institution between April 2017 and June 2018. All cases were done by operators who have been using AI guidance for AF ablations since November 2014, and have each performed over 200 such cases.

### 2.2 Compliance with ethical standards

Each patient provided written informed consent prior to the procedure. Outcome data were extracted from an institutional review board‐approved registry. The study was approved by the institutional ethics committee and performed in accordance with the ethical standards as laid down in the 1964 Declaration of Helsinki and its later amendments or comparable ethical standards.

### 2.3 AF ablation procedure

All procedures were performed under general anesthesia (GA) unless the patient expressed a desire to avoid GA in which case conscious sedation was used. Anticoagulants, either Vitamin K antagonist or non‐Vitamin K equivalents, were uninterrupted. Femoral venous access was performed under ultrasound guidance. A single transseptal puncture was performed using fluoroscopic guidance with additional pressure monitoring. Intravenous unfractionated heparin boluses were administered throughout the procedure to maintain an activated clotting time of greater than 300 seconds. Electric cardioversion was performed if patients were in AF to restore sinus rhythm prior to LA mapping. Respiratory artifact was eliminated (AccuResp Module; Biosense‐Webster, Inc) in both groups. A three‐dimensional navigation system (CARTO 3, Biosense Webster, Inc) was used to create a three‐dimensional (ConfiDENSE) electroanatomical voltage map of the LA during constant pacing from proximal coronary sinus (CS) at 600 ms, with integration with a computed tomography or magnetic resonance imaging reconstruction of the LA (CartoMerge; Biosense Webster, Inc) where available. In particular, the ConfiDENSE LA voltage map was created during atrial pacing at 600 ms with a Thermocool SmartTouch irrigated tip CF‐sensing ablation catheter (Biosense Webster, Inc), guided by the directional force vector.

All PVI procedures were performed with point‐by‐point RF wide‐area circumferential ablation (WACA) using a Thermocool SmartTouch irrigated tip CF‐sensing ablation catheter (Biosense Webster, Inc). A deflectable sheath (Agilis NxT steerable introducer; Abbott, Inc) was used in all cases. RF ablations were delivered at least 10 mm outside the PV ostia, in a power‐control mode with temperature limited to 48°C, maximal power output of 35‐40 W, targeted CF 5‐40 g, and saline irrigation rate of 17 mL/min. All ablations were performed with adherence to CLOSE protocol.^7^ Automated lesion tagging (VisiTag^TM^, Biosense Webster, Inc) was used for RF lesion marking, with a lesion display size of 3 mm. The VisiTag^TM^ settings were: minimum time 5 seconds, maximum range 3 mm, minimum CF 5 g, force‐over‐time 30%. ITD was set ≤6 mm. Each RF lesion was delivered guided by AI ≥ 400 at posterior wall and ≥550 at anterior wall. First‐pass isolation was defined as PVI of both ipsilateral veins after wide‐antral encirclement at first attempt. In cases performed under GA, esophageal temperature was monitored continuously, and RF delivery terminated if the esophageal temperature reached 38.5°C, or earlier at the operator's discretion.

All patients had PVI‐only in this study unless they had documented cavo‐tricuspid‐isthmus (CTI)‐dependent atrial flutter, in which case they also received CTI ablation. No additional ablation was performed in any patient.

**FIGURE 1:**
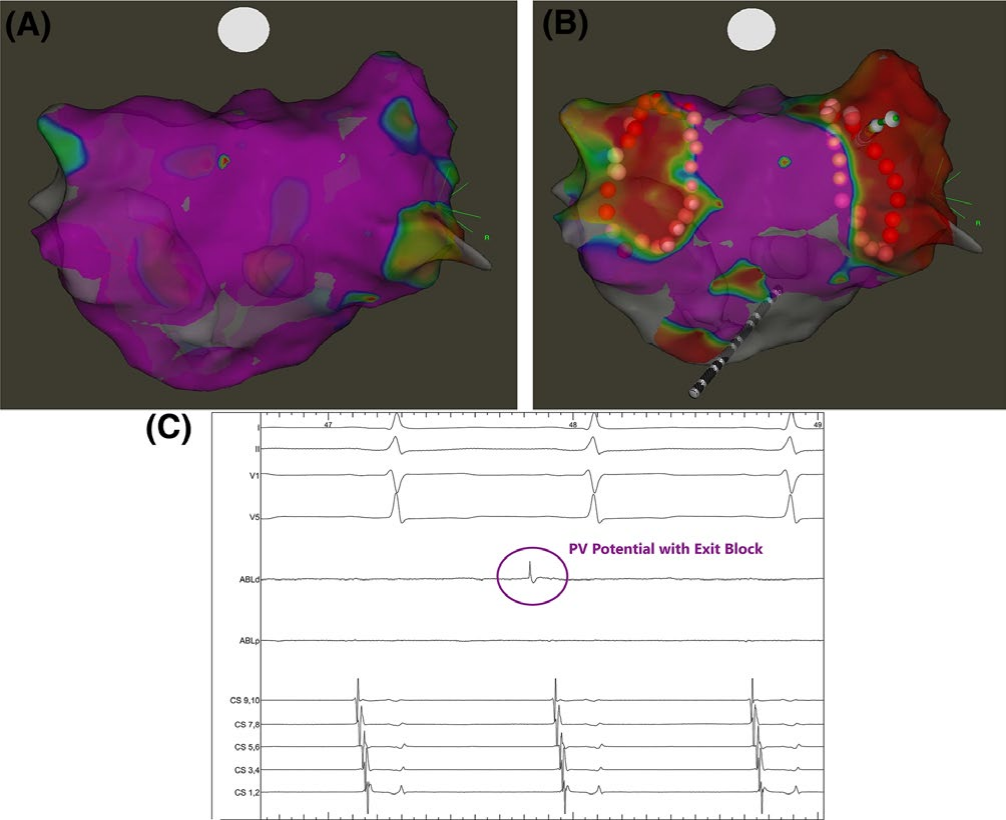
CARTO‐guided electroanatomical maps (posterior view) pre‐ (A) and post‐WACA (B). C, illustrated a pulmonary vein (PV) ectopy with exit block when the ablation catheter was placed at the PV ostium following wide‐area circumferential ablation

**FIGURE 2:**
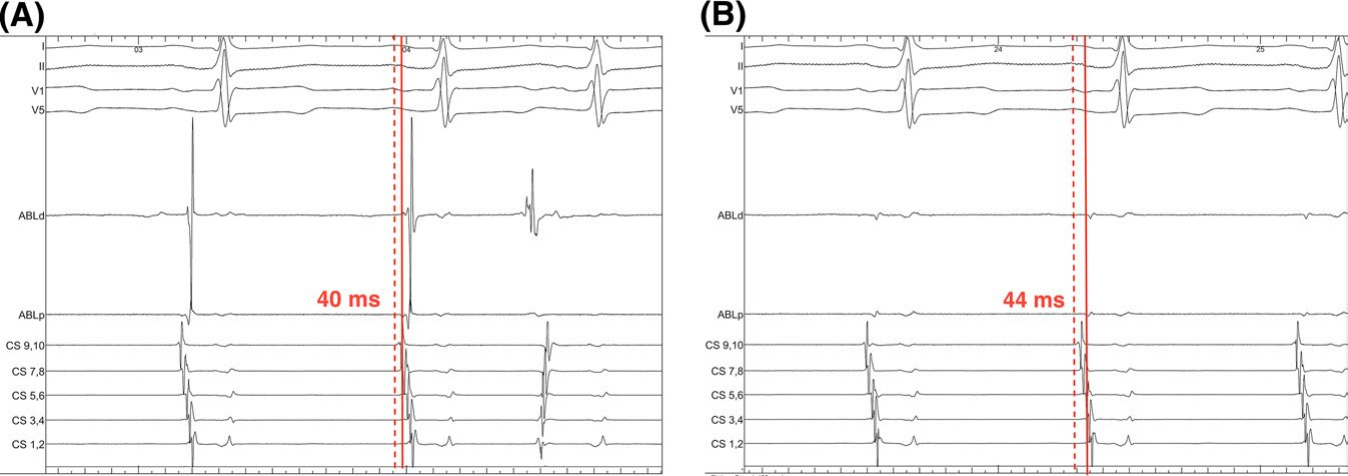
Differentiation of far‐field signals from left atrial (LA) appendage. Timings of CS9,10 to signals at distal bipole of ablation catheter were similar when ablation catheter was placed on the LA appendage side of the ridge (A) and on the left superior pulmonary vein side (B)

**FIGURE 3:**
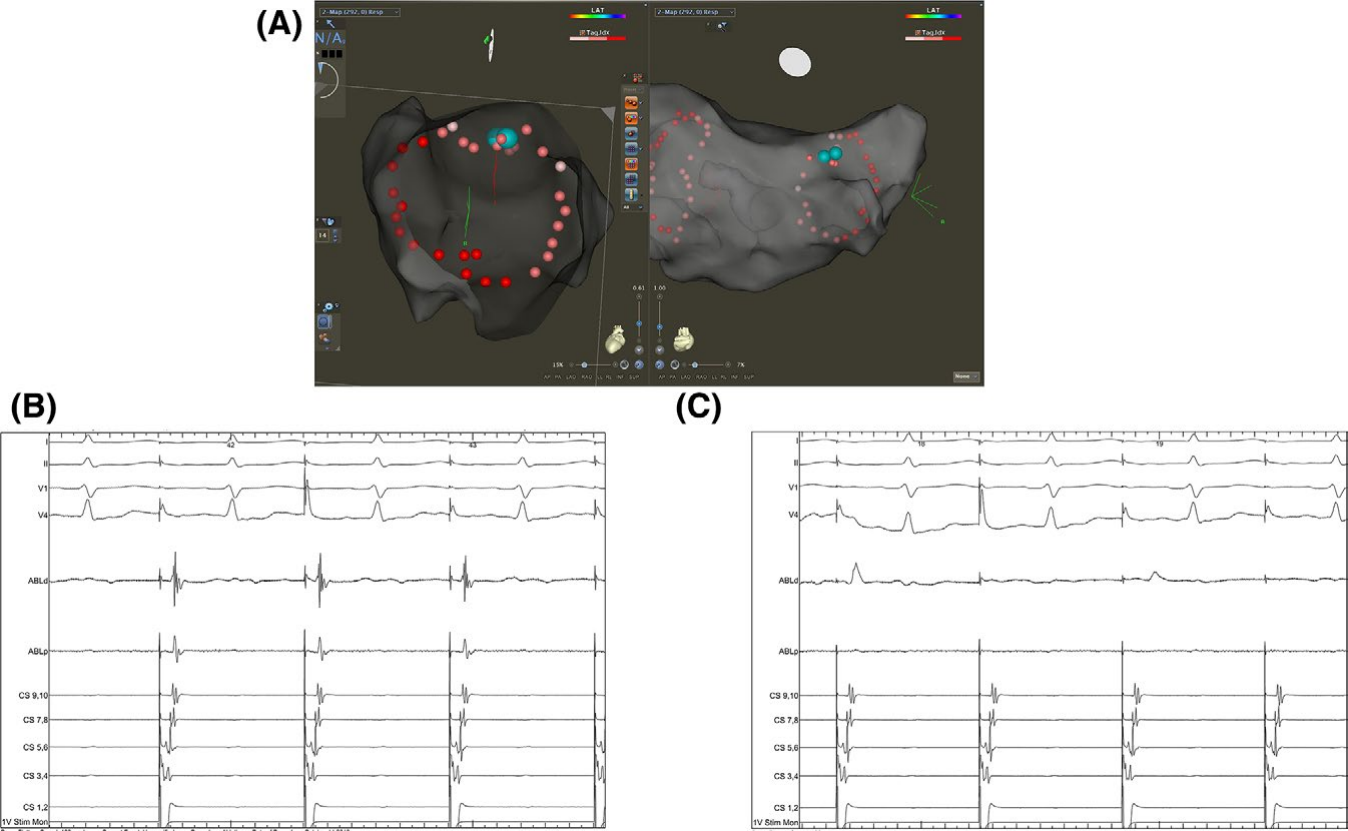
Identification of pulmonary vein (PV) entrance conduction gap in a right superior PV. When the ablation catheter was placed in the lesion set gap at the right posterior carina (blue orbs; A), timings of CS1,2 to signals at distal bipole of ablation catheter were shortest (B). Ablation in this region led to successful pulmonary vein isolation (C)

Following WACA, LA electroanatomical mapping was performed with the ablation catheter placed just inside the WACA to validate entry block and to assess for sites of reconnection (Figure 1A,B). Further assessment of exit conduction block was performed by placing the ablation catheter into each of the four PV ostia and eliciting PV ectopy by tapping the tissue (Figure 1C). If no clear PV ectopy was observed, pacing at 10mA output was performed from the distal bipole of the ablation catheter that was placed antrally at four quadrants of each WACA circle with CF ≥ 10 g. Exit block was demonstrated as local capture with no exit from the WACA as visualized on the CS electrodes. The ablation catheter was placed on the anterior and posterior aspect of each carina to confirm entry and exit block. In the presence of a possible far‐field signal from the LA appendage, the signal in question was verified by comparison of timings of proximal CS electrodes to ablation signal when the ablation catheter was placed in the LA appendage or in the left superior PV (Figure 2). Similarly, far‐field signal from the superior vena cava could be differentiated by the timing of the signal with the first half of the surface P‐wave when the ablation catheter was in the right superior PV. If residual PV signals were identified, the site(s) of breakthrough were identified by measuring conduction times along the WACA and at the carina during CS pacing (Figure 3). If PVI was not achieved with ablation of 2 or more distinct sites along the WACA, a CMC was taken to localize the breakthrough site(s).

### 2.4 Patient follow‐up and clinical outcomes

Patient follow‐up was conducted as per local standard practice with clinical reviews at 3, 6, and 12 months with mandatory 12‐lead electrocardiograph (ECG) at each follow‐up visit, and supplementary symptom‐driven ambulatory monitoring if required. All patients discontinued their antiarrhythmic medications (Class I and III) following the initial blanking period of 3 months. The rates of recurrence of atrial arrhythmia, defined as >30 seconds of any atrial arrhythmias (AF, flutter, or atrial tachycardia) with ECG documentation after the initial 3‐month blanking period, were captured over the 1‐year follow‐up period. For survival analysis (survival free from atrial arrhythmia), patients were censored at 1 year if no atrial arrhythmia had occurred or at the date of last follow‐up.

### 2.5 Statistical analysis

Continuous variables were expressed as mean ± SD for normally distributed data, and as median with interquartile range (IQR, 25^th^‐75^th^ percentiles) if not normally distributed. All statistical analysis was performed using SPSS (version 24; IBM Corp.). Graphs were drawn using GraphPad Prism (version 8; GraphPad Software Inc).

## 3 RESULTS

A total of 67 consecutive patients underwent RF PVI procedures for paroxysmal and persistent AF during the study period using single‐catheter approach.

### 3.1 Patient demographics

Table 1 illustrates that baseline demographics including age, gender distribution, echocardiographic parameters, relevant comorbidities, AF subtypes, and preprocedural antiarrhythmic medications. Thirty patients (44%) had paroxysmal AF, and 37 (56%) had persistent AF. No patient had long‐standing persistent AF. The mean CHA_2_DS_2_‐VASc score was 1.6 ± 1.3 and the mean LA size was 4.2 ± 0.6 mm, suggestive of patients who did not have advanced AF substrate.

**TABLE 1:** Patient demographics

Parameter	Study group (n = 67)
Male gender, n (%)	50 (75)
Age (y)	63 ± 10
CHA_2_DS_2_‐VASc score	1.6 ± 1.3
Echocardiography	
LV function (% EF)	57 ± 6.3
LA diameter (cm)	4.2 ± 0.6
PAF:PeAF	30:37
Antiarrhythmics	
None	12/ 67
Beta blockers	44/67
Class I drugs	7/67
Class III drugs	12/67

Note

Abbreviations: LA left atrium; LV left ventricle; PAF paroxysmal atrial fibrillation; PeAF persistent atrial fibrillation.

### 3.2 Procedural details

Table 2 provides a summary of the procedural data. Most procedures were performed under general anesthesia (65 of 67 [97%]). In 58 (86.6%) cases, first‐pass PVI was achieved after initial encirclement of PV antra. In the nine cases requiring touch‐up ablations in the study group, mapping with the ablation catheter alone during CS pacing was successful in identifying gaps in eight (88.9%) cases. Each of these patients had only one gap; on the posterior aspect of the right intervenous carina in six patients, and on the anterior aspect of the left intervenous carina in two patients. One patient (1.5%) required the use of a CMC. In this patient, multiple touch‐up ablations were required for both right and left PVs, eventually requiring a linear ablation across both intervenous carinae with concurrent use of CMC to achieve PVI.

**TABLE 2:** Procedural details

Parameter	Study group
General anesthesia, (%)	65 (97)
Procedure duration (min)	147.9 ± 32.4
Ablation time (min)	27.5 ± 5.7
Fluoroscopy time (min)	7.8 ± 5.4
Radiation dosage (mGy.cm^2^)	806.1 ± 539.5
First‐pass PVI rate, (%)	58 (86.6)
Complications	
Minor^a^	1/67^c^
Major^b^	0/67

Note

Abbreviation: PVI, pulmonary vein isolation.

^a^ No delay in hospital discharge.

^b^ Delay in hospital discharge.

^c^ Urinary retention.

The RF ablation time was 27.5 ± 5.7 minutes. The mean procedure duration was 147.9 ± 32.4 minutes, mean fluoroscopy time was 7.8 ± 5.4 minutes, and mean radiation exposure was 806.1 ± 539.5 mGy cm^2^. These were significantly shorter than the mean procedure time, fluoroscopy time, and radiation exposure seen with our AI guided AF ablation procedures using a CMC catheter^5^: 175 ± 31, 11.9 ± 7.7 minutes, and 1,656 ± 1,425 mGy cm^2^, respectively, *P* < .001 for all.

There were no major procedural complications seen.

### 3.3 Follow‐up and clinical outcomes

The mean follow‐up of the study was 390 [IQR 138] days.

**FIGURE 4:**
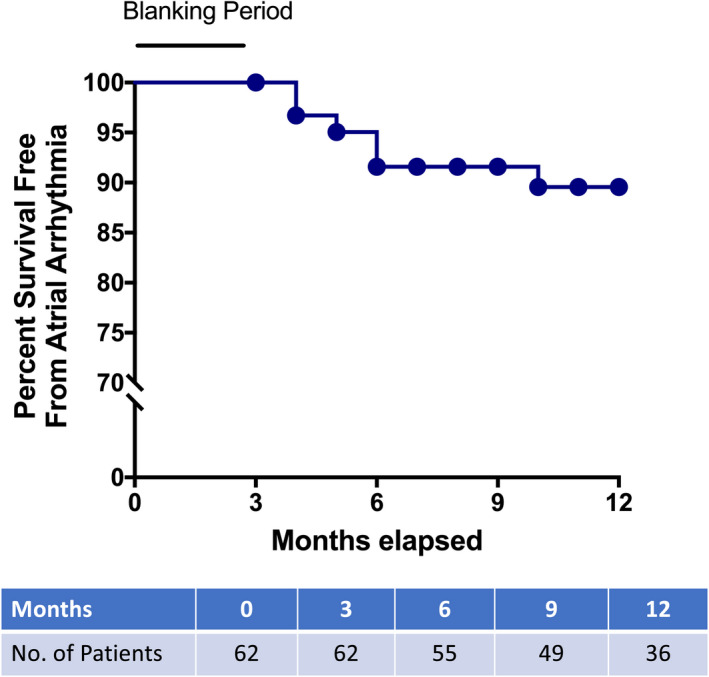
Kaplan‐Meier curves of atrial arrhythmia‐free survival with circular mapping catheter‐free atrial fibrillation ablation approach during the 12‐mo follow‐up period

Arrhythmia recurrence, defined as the absence of AF/atrial flutter/atrial tachycardia following a 3‐month blanking period after a single procedure off antiarrhythmic medications, was seen in seven (10.4%) patients. At 12 months, Kaplan‐Meier survival analysis showed 89.6% of patients free from atrial arrhythmias in the study (Figure 4).

### 3.4 Cost implications of the single‐catheter approach

The cost of a CMC catheter, and a transseptal sheath at our institution are £625 and £90, respectively. These costs were saved in 66 of the 67 cases to give a total cost saving of £47, 190 compared to our traditional PVI approach that utilizes two transseptal sheaths and a CMC.

## 4 DISCUSSION

To our knowledge, this study represents the first report on the use of a CMC‐free single‐catheter technique for RF PVI guided by the CLOSE protocol. Using this approach, we demonstrated the following findings: (a) a CMC‐free approach is feasible in the vast majority of cases of PVI with an efficient and safe procedural workflow with short procedure duration and ablation times; (b) almost 90% of circles are isolated with first‐pass isolation alone, and the overwhelming majority of gaps are seen on the intervenous carinae; (c) this “minimalist” approach results in an excellent outcome of freedom from atrial arrhythmias at medium‐term follow‐up, and (d) significant cost savings by virtue of the exclusion of a CMC and the second transseptal sheath.

### 4.1 The reliability of assessment of conduction block with a single‐catheter approach

Since the discovery of PV triggers, successful electrical isolation of the PVs has been regarded as the cornerstone of all AF ablations.^1^ The use of CMC has since been the widely accepted means for assessment of conduction breakthrough from the PVs. As the PVI procedure evolved over the years, the ablation level shifted from the PV ostia to the LA antra to increase the efficiency of PVI and to reduce the risk of PV stenosis. The conventional CMC placement at the PV ostia has made the earliest potentials recorded by the CMC less relevant and reliable because of the variable anatomy of the veno‐atrial junction contributing to both anatomical and functional conduction blocks.^9^ It is therefore arguable that for first‐time PVI procedures, the ablation catheter may provide greater flexibility and precision in maneuvering around the antral region for assessment of conduction block. Previous studies have demonstrated that single‐tip catheter was capable of assessing PVI based on the loss of pace capture along the lesion sets.^10^, ^11^, ^12^ Although this method is highly predictive of PVI, this method could be confounded by several issues. First, poor catheter‐tissue contact may masquerade as loss of pace capture although this could be circumvented by ensuring CF to be at least 10 g.^12^ Even with adequate CF, this however failed to address the challenging issue of obtaining convincing near‐field capture because of the oversaturation of pacing artifact obliterating the antral potentials. Second, infrequently epicardial fibers may exist to provide “concealed” conduction breakthroughs within the antrum typically at the carina level, thereby bypassing the endocardial ablation line to provide a false negative phenomenon. However, the use of CMC in conventional workflow is not infallible to these “concealed” antral PV conduction, often necessitating high‐density mapping to resolve this issue.^13^ In this study, we elected to validate PVI not only by collecting antral voltage maps pre‐ and postablation but also by demonstrating exit block of PV ectopy thereby obviating the need for pacing adjacent to the PV sleeve unless we failed to elicit PV ectopy during the manipulation of the ablation catheter within the PV ostium. Using this method, we managed to avoid the aforementioned pitfalls.

The two‐catheter setup, however, does allow for differentiation of the far‐field LA appendage potentials without resorting to limiting the pacing output.^14^ Although far‐field potentials from LA appendage and superior vena cava could potentially pose diagnostic challenges in validating PVI especially at the anterior aspects of the left and right superior PV, we find that the comparison of timing measurements during CS pacing from pacing spike to either the signals of ablation catheter in LA appendage or with the surface P‐wave would aid in addressing the issue without resorting to complex pacing maneuvers (Figure 2). Importantly, if residual PV gaps were present following initial delivery of PV encirclement lesion sets, judicious timing measurements between pacing spikes during CS pacing and the PV signals at the roving ablation catheter with the aid of CARTO‐VisiTag^TM^ and ITD (Figure 3A) allows precise localization of the earliest entrance conduction (Figure 3B) with successful PVI (Figure 3C). It is important to note that the majority of residual conduction after a CLOSE‐guided PVI occurs along the unablated intervenous carinal tissue. This knowledge helps to quickly home in on the likely sites of residual conduction in cases where first‐pass isolation has not been achieved.

### 4.2 A streamlined approach of AI and ITD

Efficient PVI relies on RF delivery of contiguous and safe LA antral lesions with adequate transmurality. The advent of CF‐sensing catheters paved the way for optimizing lesion depth and AF ablation outcome.^15^ AI was developed to incorporate CF, time and power in a weighted formula, and has emerged as a reliable surrogate marker for the durability of PVI and more importantly a strong correlation with clinical outcomes.^4^, ^5^, ^16^ In spite of the use of force‐sensing catheters, ablation outcomes remained suboptimal.^17^, ^18^ This may be accountable by the lack of contiguity of ablation lesions within the RF encirclement even in the context of adequate CF.^19^, ^20^ Indeed, the marriage of regimental AI applications (AI ≥ 550 at anterior wall, AI ≥ 400 at posterior wall) and strict ITD criteria (≤6 mm), that is, the CLOSE protocol, has yielded excellent first‐pass PVI rates and high single‐procedure freedom from atrial arrhythmia at 1 year.^8^

In one study, loss of capture along the PV lesion sets translated as superior AF freedom at 18 months when compared to traditional demonstration of conduction block using CMC.^21^ Furthermore, the technique of pacing for unexcitability has been shown to reliably identify sites of dormant conduction including sites not identifiable by adenosine administration.^22^ For operators wishing to check for PV dormant conduction after initial PVI, this then abolishes the extra step of adenosine administration using the single‐catheter approach.

Pambrun et al^12^ assessed the feasibility of single‐catheter PVI with CF‐sensing catheters, and our study extends this to an AI‐guided CLOSE protocol approach. Indeed, adherence to the CLOSE protocol with a single‐catheter setup yielded a near 90% freedom from atrial arrhythmias at 1‐year follow‐up similar to the clinical outcome in that study. In contrast, we highlighted the pitfalls of the single‐catheter technique, acknowledging and addressing the issue of far‐field signals for verification of PVI. We also provided a simpler way of demonstrating PV exit block through eliciting a nonconducted PV ectopy as an adjunct to pacing within all four quadrants of the PV as advocated by Pambrun et al.

### 4.3 Safety and cost‐effectiveness of a minimalistic approach

The inherent risk of several complications of AF ablation can potentially be reduced with a single‐catheter approach. The avoidance of second sheath/catheter manipulation and limiting the number of transseptal punctures may reduce the risk of tamponade. The potential of cerebral embolism when exchanging CMC with ablation catheter through a single transseptal sheath strengthens the case for a single‐catheter, single sheath approach.^23^ Although a rare complication, the risk of CMC entrapment in the mitral valve apparatus remain another concern, adding further to the safety profile of the single‐catheter approach.^24^

Healthcare economic principles dictate conscientious use of consumables and an efficient AF ablation workflow. This study demonstrated the cost saving implication of the single‐catheter approach in terms of lower costs of consumables.

### 4.4 Limitations

Several limitations need to be acknowledged in this study. First, patient recruitment into this study is nonrandomized, although consecutive in nature. Second, the differentiation of PV potentials from far‐field potentials, as well as localization of residual gap(s) after first‐pass isolation require patients to be in sinus rhythm. Therefore, for accurate PVI validation using the single‐catheter approach, patients in AF need to be electrically cardioverted. Finally, it is improbable to ascribe our excellent ablation outcome at 12 months to the single‐catheter setup per se. More likely than not, it is strict adherence to the CLOSE protocol that permits this streamlined single‐catheter approach, thereby obviating the need of a CMC to achieve similar, if not better outcomes. This may not be replicable by relatively inexperienced operators.

## 5 CONCLUSION

A streamlined single‐catheter workflow for contiguous AI‐guided AF ablation for PVI is safe and efficient, while delivering cost savings when compared to the conventional two‐catheter ablation setup. This approach is also associated with excellent ablation outcome at 12 months.

## CONFLICT OF INTEREST

DG received research funding from Biosense Webster and participated on research grants supported by Johnson and Johnson.

## ETHICAL APPROVAL

All procedures performed in studies involving human participants were in accordance with the ethical standards of the institutional research board (IRB number: LHCH‐R&D 1117, approved on 18th March 2017) and with the 1964 Helsinki declaration and its later amendments or comparable ethical standards.
